# Anandamide Suppresses Proliferation and Cytokine Release from Primary Human T-Lymphocytes Mainly via CB_2_ Receptors

**DOI:** 10.1371/journal.pone.0008688

**Published:** 2010-01-14

**Authors:** Maria Teresa Cencioni, Valerio Chiurchiù, Giuseppina Catanzaro, Giovanna Borsellino, Giorgio Bernardi, Luca Battistini, Mauro Maccarrone

**Affiliations:** 1 European Center for Brain Research (CERC)/Santa Lucia Foundation, Rome, Italy; 2 Department of Biomedical Sciences, University of Teramo, Teramo, Italy; 3 Department of Neurosciences, University of Rome “Tor Vergata”, Rome, Italy; INSERM U862, France

## Abstract

**Background:**

Anandamide (AEA) is an endogenous lipid mediator that exerts several effects in the brain as well as in peripheral tissues. These effects are mediated mainly by two types of cannabinoid receptors, named CB_1_R and CB_2_R, making AEA a prominent member of the “endocannabinoid” family. Also immune cells express CB_1_ and CB_2_ receptors, and possess the whole machinery responsible for endocannabinoid metabolism. Not surprisingly, evidence has been accumulated showing manifold roles of endocannabinoids in the modulation of the immune system. However, details of such a modulation have not yet been disclosed in primary human T-cells.

**Methodology/Significance:**

In this investigation we used flow cytometry and ELISA tests, in order to show that AEA suppresses proliferation and release of cytokines like IL-2, TNF-α and INF-γ from activated human peripheral T-lymphocytes. However, AEA did not exert any cytotoxic effect on T-cells. The immunosuppression induced by AEA was mainly dependent on CB_2_R, since it could be mimicked by the CB_2_R selective agonist JWH-015, and could be blocked by the specific CB_2_R antagonist SR144528. Instead the selective CB_1_R agonist ACEA, or the selective CB_1_R antagonist SR141716, were ineffective. Furthermore, we demonstrated an unprecedented immunosuppressive effect of AEA on IL-17 production, a typical cytokine that is released from the unique CD4+ T-cell subset T-helper 17.

**Conclusions/Significance:**

Overall, our study investigates for the first time the effects of the endocannabinoid AEA on primary human T-lymphocytes, demonstrating that it is a powerful modulator of immune cell functions. In particular, not only we clarify that CB_2_R mediates the immunosuppressive activity of AEA, but we are the first to describe such an immunosuppressive effect on the newly identified Th-17 cells. These findings might be of crucial importance for the rational design of new endocannabinoid-based immunotherapeutic approaches.

## Introduction

Anandamide (*N*-arachidonoylethanolamine, AEA) is an endogenous arachidonic acid derivative which functionally belongs to the family of endocannabinoids (ECs), an evergrowing class of lipid mediators isolated from brain and peripheral tissues [1], [2]. AEA exerts its action through binding and activation of cannabinoid receptors, CB_1_R and CB_2_R [3]. These receptors are differentially distributed among cells, with CB_1_R being preferentially expressed in the brain and in other neural tissues, whilst CB_2_R is found mainly in cells of the immune system [4], [5]. The presence of endogenous ligands for the cannabinoid receptors has increased awareness of the potential importance of endocannabinoids in regulating and fine-tuning several processes, including neuroprotection [6] and immune responses [7]. When stimulated, immune cells up-regulate particular receptors or express new ones. This scenario has been demonstrated also for CBRs, since several stimuli have been reported to differently modulate both cannabinoid receptor subtypes in immune cells, like mouse splenocytes, Jurkat T-cells, mouse macrophages and microglia, as well as human tonsillar B cells and dendritic cells [8]. In addition, it has been shown that chronic marijuana smoking increases CB_1_R and CB_2_R mRNA in peripheral blood mononuclear cells [8]. Modulation of CBRs gives support to a regulatory role of endocannabinoids in immune responses, however the molecular details of this activity have yet to be fully elucidated. So far, AEA has been detected in different immune cells, including dendritic cells, macrophages, microglia, and lymphocytes [9]. Moreover, activation of lymphocytes and other immune cells causes a rapid and robust increase in AEA levels, further supporting a role for AEA in immune regulation [10]. For instance, AEA suppresses LPS-induced nitric oxide production by mouse peritoneal macrophages [11], and LPS-induced cytokine mRNA expression in rat microglial cells [12]. In addition, AEA decreases mitogen-induced T and B cell proliferation [13], suppresses CD8 T lymphocyte migration [14], and induces apoptosis in macrophages [15] and dendritic cells [16]. In the present study we sought to better define the role of AEA in modulating immune functions of primary human T cells, with a special focus on the key regulators of autoimmune inflammation: Th17 lymphocytes [17].

## Materials and Methods

### Cell preparation

Peripheral blood mononuclear cells (PBMCs) from healthy donors were isolated by Ficoll-hystopaque gradient centrifugation (Pharmacia, Uppsala, Sweden) and cultured at 10^6^ cells/ml in RPMI 1640 medium supplemented with 10% (vol/vol) heat-inactivated human serum (Life Technologies, Grand Island, NY), 2 mM L-glutamine, 20 mM HEPES [*N*-2-hydroxyethylpiperazine-*N*'2-ethanesulfonic acid] and 10 U/ml penicillin and streptomycin (Life Technologies). Using the Pan T cell Isolation Kit (Miltenyi Biotec, Germany), highly purified human CD3^+^-T cells were isolated by depletion of CD3^−^ non-T cells (negative selection). The purity of the enriched CD3^+^-T cells was evaluated by Flow Cytometry and was consistently above 90%.

### Immunoblotting of Cannabinoid Receptors

Purified human T lymphocytes were lysed with RIPA Buffer (1% Nonidet P40, 0.1% sodium dodecyl sulphate (SDS), 0.5% sodium deoxycholate, 0.1% protease inhibitor cocktail, 50 mM sodium fluoride and 100 µM sodium orthovanadate in phosphate-buffered saline, PBS), and centrifuged for 30 min at 18000 x *g* at 4°C. The supernatants were recovered and the protein concentration measured using the Bradford assay. The expression of CB_1_R and CB_2_R was assessed by Western blotting. Cell homogenates were subjected to 10% SDS-PAGE (50 µg/lane) under reducing conditions, then gels were electroblotted onto 0.45-µm nitrocellulose filters (Bio-Rad, Hercules, CA) and were immunoreacted with anti-CB_1_R (1∶250) or anti-CB_2_R (1∶500) polyclonal antibodies (Cayman Chemical Co., Ann Arbor, MI), or with anti-β-actin monoclonal antibody (1∶5000, Bio-Rad). GAR-AP (1∶2000, Santa Cruz Biotechnologies, Santa Cruz, CA) or GAM-AP (1∶10000, Bio-Rad) were used as second antibody. The specificity of anti-CB_1_ or anti-CB_2_ antibodies was ascertained by preincubating 1 µg of each antibody with 10 µg of the specific blocking peptide (Cayman Chemical Co., Ann Arbor, MI).

### qRT-PCR Analysis

RNA was extracted from purified human T-lymphocytes using the RNeasy extraction kit (Qiagen, Crawley, UK), as suggested by the manufacturer. Quantitative real time reverse transcriptase (qRT)-PCR assays were performed using the Super-Script III Platinum two-step qRTPCR kit (Invitrogen). One µg of total RNA was used to produce cDNA with 10 units/µl SuperScript III reverse transcriptase, in the presence of 2 units/µl RNaseOUT, 1.25 µM oligo(dT)20, 1.25 ng/µl randomhexamers, 5 mM MgCl_2_, 0.5 mM dNTP mix, and diethyl pyrocarbonate-treated water. The reaction was performed using the following qRT-PCR program: 25°C for 10 min, 42°C for 50 min, 85°C for 5 min, then after addition of 0.1 unit/µl of *Escherichia coli* RNase H, the product was incubated at 37°C for 20 min. The target transcripts were amplified by means of an ABI PRISM 7700 sequence detector system (Applied Biosystems, Foster City, CA) using the following primers: human *CB_1_R* F1 (5′-CCTTTTGCTGCCTAAATCCAC-3′) and human *CB_1_R* R1 (5′-CCACTGCTCAAACATCTGAC-3′); human *CB_2_R* F1 (5′-TCAACCCTGTCATCTATGCTC-3′) and human *CB_2_R* R1 (5′-AGTCAGTCCCAACACTCATC-3′). β-Actin was used as housekeeping gene for quantity normalization. One µl of the first strand of cDNA product was used for amplification (in triplicate) in a 25 µl reaction solution, containing 12.5 µl of PlatinumSYBRGreenqPCRSuper-Mix UDG (Invitrogen) and 10 pmol of each primer. The following PCR program was used: 95°C for 10 min, 40 amplification cycles at 95°C for 30 s, 56°C for 30 s, and 72°C for 30 s.

### Confocal Microscopy

Purified T-lymphocytes were seeded on Chamberslides (Lab-Tek) at 1×10^5^ cells/well and were stimulated or not with anti-CD3 and anti-CD28 polyclonal antibodies for 24 h. Cells were then fixed and were stained with anti-CB_1_R or anti-CB_2_R specific antibodies. The surface localization of cannabinoid receptors was visualized by confocal microscopy (Leica TCS SP), performing image acquisition through the LAS AF program (Leica).

### [^35^S]GTPγS Assay

Resting or anti-CD3/anti-CD28-activated purified human T-lymphocytes (2.5×10^6^ cells/test) were incubated in the presence or absence of the synthetic agonist of cannabinoid receptors CP55.940 (2.5 µM), for 60 min at room temperature in 20 mM HEPES, 100 mM NaCl, 5 mM MgCl_2_, 0.2% (w/v) BSA buffer (pH 7.6), supplemented with 20 µg/ml saponin (Sigma Chemical Co., St. Louis, MO), 25 µM GDP (Sigma) and 0.3 nM [^35^S]GTPγS (Perkin Elmer Life Sciences). Nonspecific binding was determined in the absence of agonist and in the presence of 10 µM unlabeled GTPγS (Perkin Elmer Life Sciences), as reported [18].

### Flow Cytometry Analysis

Freshly isolated PBMCs were left untreated or were pre-treated with 2.5 µM AEA (Sigma); cells were stimulated with plate-bound anti-CD3 (clone T3D; 5 µg/ml) and soluble anti-CD28 antibody (2 µg/ml) (e-Bioscience) for 48 hours. Cells were then washed twice and stained with anti-CD3, anti-CD8, anti-CB1R and anti-CB2R antibodies in PBS, supplemented with 0.5% FCS and 0.02% NaN_3_. Surface receptors expression was analysed by FACS (FACSCanto, Becton-Dickinson).

### Antibodies

Cells were stained for Cytometry analysis using the antibodies detailed in **Table 1**.

**Table 1:** Details of the antibodies used in the study.

ANTIBODY	MANUFACTURER	DILUTION
**CB_1_R**	Affinity Bioreagents	1∶200
**CB_2_R**	Cayman Chemicals	1∶200
**CD3 (APC-Alexa Fluor750)**	eBioscience	1∶40
**CD8 (Pe-Cy7)**	Beckman Coulter Inc.	1∶40
**Goat-anti-rb IgG (Alexa Fluor 633)**	Invitrogen, Molecular Probes	1∶200
**TNF-α-APC**	eBiosciences	1∶40
**IFN-γ-PE**	BD Biosciences Pharmigen	1∶40
**IL-17-PE**	eBiosciences	1∶20

### Proliferation Assay

T-cells were plated at 1×10^5^ cells/well and were treated or not with 2.5 µM AEA and selective CB_1_R or CB_2_R agonists, ACEA (arachidonoyl-2-chloroethylamide) and JWH-015 ((2-methyl-1-propyl-1H-indol-3-yl)-1-napthalenylmethanone) respectively, or antagonists (SR141716 (*N*-(piperidin-1-yl)-5-(4-chlorophenyl)-1-(2,4-dichlorophenyl)-4-methyl-1H-pyrazole-3-carboxamide hydrochloride) [SR1], and SR144528 (*N*-(1,S)-endo-1,3,3-trimethyl-bicycle(2,2,1)heptan-2-yl)-5-(4-chloro-3-methylphenyl)-1-(4 methylbenzyl)-pyrazole-carboxamide) [SR2], respectively). CBRs antagonists were used in combination with their respective agonist, according to the experimental conditions. After treatments, cells were plate-bound and then were stimulated with purified anti-CD3 (T3D; 5 µg/mL) and soluble anti-CD28 antibody (2 µg/mL; e-Bioscience). Proliferation of AEA-pretreated PBMCs was assessed by monitoring carboxyfluorescein diacetate (CFDA; Molecular Probes) dilution, after stimulation with CD3/CD28. Cell divisions were followed by flow cytometry every two days for one week. All flow cytometric data were analyzed with Flowjo software (Treestar, Ashland, OR). Proliferation was also assessed by [^3^H]thymidine incorporation, labelling unstimulated or stimulated cells with 1 µCi/well [^3^H]thymidine (Perkin Elmer) after 4 days of culture. After further 12 hours, cell proliferation was determined using a beta-plate reader (Tomtec, Gaithersburg, MD), according to the manufacturer's instructions.

### ELISA

T cells were plated at 1×10^5^cells/well and were left untreated or were pre-treated with 2.5 µM AEA, or with JWH-015, ACEA, SR1 or SR2, each used at 1.0 µM. Each antagonist was used in combination with its respective agonist, according to the experimental conditions. After treatments, cells were plate-bound and then were stimulated with purified anti-CD3 (clone T3D; 5 µg/ml) and soluble anti-CD28 antibody (2 µg/ml; e-Bioscience). Cells were maintained at 37°C for 24 hours and then supernatants were collected. Cytokines were determined by a standard 2-site sandwich enzyme-linked immunosorbent assay (ELISA), using enhanced protein-binding ELISA plates (Nunc Maxisorp; Nunc Maxi, Roskilde, Denmark). Antibody for IL-2 was purchased from Roche, while antibodies for TNF-α and IFN-γ were purchased from Pierce Endogen.

### Intracellular Staining

Freshly isolated PBMCs were pre-treated with 2.5 µM AEA and/or with selective CB_1_R or CB_2_R agonists or antagonists, and then stimulated with 1 µM phorbol 12-myristate 13-acetate (PMA, Sigma) and 10 µM ionomycin (Sigma) for 6 hours, in order to allow cytokines synthesis. Each antagonist was used in combination with its respective agonist, according to the experimental conditions. Release of cytokines was inhibited by adding 1 µg/ml brefeldine A (Sigma) 4 hours before the end of stimulation. At the end of the incubation, cells were washed twice and were stained with anti-CD3 and anti-CD8 antibodies in PBS, supplemented with 0.5% FCS and 0.02% NaN_3_. Cells were fixed with 4% paraformaldehyde (Sigma) for 10 min at room temperature. Cells were stained intracellulary with anti-TNFα-APC, anti-IFNγ-PE, or anti-IL17-PE and analysed on the flow cytometer.

### Statistics

Statistical analysis was performed by Prism 4 (GraphPAD Software for Science, San Diego, CA), using paired Student's *t*-test and assuming an unequal variance with 95% confidence levels. Only p values <0.05 were considered significant.

## Results

### Effect of AEA on CB_1_R and CB_2_R expression

**Figure 1:**
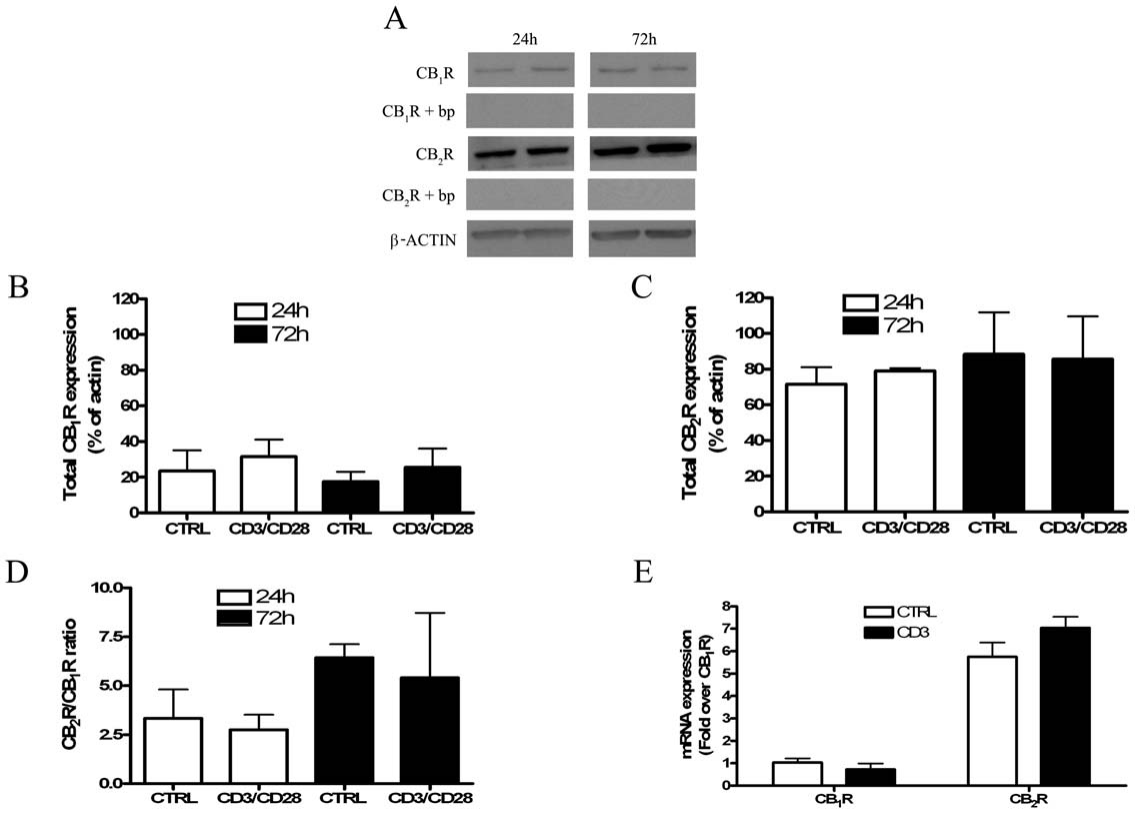
Analysis of CB_1_R and CB_2_R expression in resting and activated primary human T-lymphocytes. Freshly isolated T-lymphocytes were either left untreated or treated with 5 µg/ml anti-CD3 and 2 µg/ml anti-CD28 for the indicated periods of time (24–72 hours). **Panels A–D**: Cells were lysed and the homogenates were assayed for CB_1_R and CB_2_R protein by Western blot analysis. CB_1_R and CB_2_R protein levels were quantified by densitometric analysis, and were normalized to beta-actin. The results were expressed as total amount of receptors over 24 hours and 72 hours of stimulation (**A**, **B** and **C**) (average value of all samples) as well as CB_2_R to CB_1_R ratio (**Panel D**). Panel **E**: Cells were washed in PBS, and then mRNA was isolated and analysed by qRT-PCR using specific primers for CB_1_R and CB_2_R. Data are representative of 4 independent experiments.

**Figure 2:**
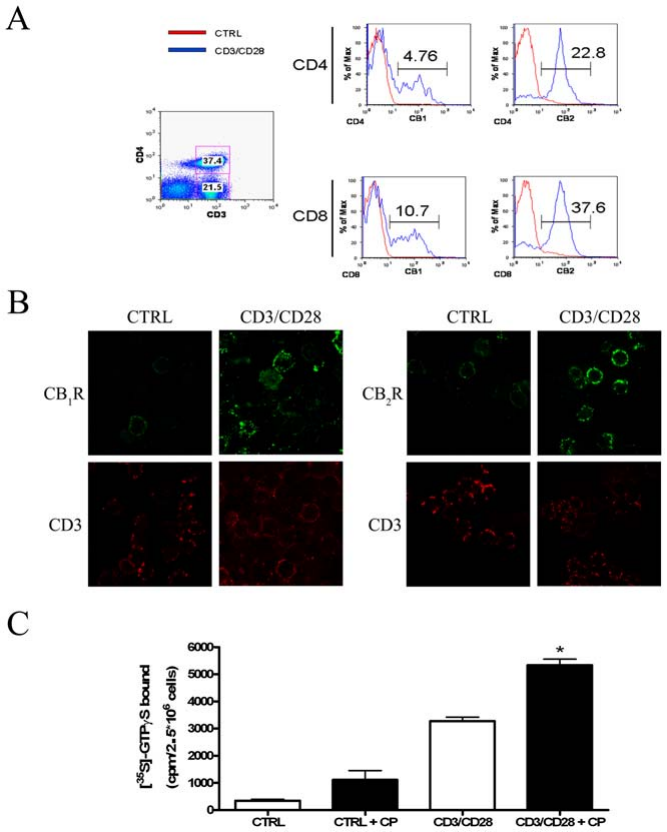
Surface expression of CB_1_R and CB_2_R on primary human T-lymphocytes. Freshly isolated human T-lymphocytes were stimulated or not with anti-CD3 and anti-CD28 for 24 hours. **Panels A–B**: Surface expression of CB_1_R and CB_2_R was reported as MIF (medium intensity of fluorescence) values on a FACS-Canto, gating for CD4^+^CD3^+^ and CD4^−^CD3^+^ cells (**A**), and by confocal microscopy staining with anti-CD3, and anti-CB_1_R or anti-CB_2_R (**B**). **Panel C**: Resting or activated T-cells were either left untreated or incubated with 2.5 µM CP55.940 (CP), 20 µg/ml saponin, 25 µM GDP and 0.3 nM [^35^S]GTPγS. The amount of [^35^S]GTPγS bound is reported as counts per min (cpm) per 2.5×10^6^ cells. * Indicates p<0.005 *versus* CD3/CD28-treated cells. Data are representative of 4 independent experiments.

Initial studies were performed to assess the modulation, if any, of cannabinoid receptors expression by purified T lymphocytes following their activation. **Fig. 1A** shows that activated T-cells express higher levels of CB_2_R than CB_1_R at two distinct experimental time points (24 h and 72 h). These immunoreactive bands were fully erased by preincubation of anti-CB_1_ and anti-CB_2_ antibodies with their specific blocking peptides, demonstrating antibody specificity (**Fig. 1A**). The pattern of CBRs expression in resting and activated T-lymphocytes can be better appreciated by means of densitometric analysis of the blots, shown in **Figs. 1B–D**. In fact, CB_2_R expression in activated cells displayed a ∼2.5-fold increase compared to that of CB_1_R. However, no significant difference could be observed between controls and anti-CD3/anti-CD28-treated cells at both experimental times. In order to further analyze CBRs expression, we quantified the mRNA levels of the receptors in resting and anti-CD3/anti-CD28-stimulated T-cells; we found that CB_2_R mRNA was ∼6-fold higher than that of CB_1_R under both conditions (**Fig. 1E**). These results led to the hypothesis of a possible re-localization of cannabinoid receptors within the cell upon activation. In order to substantiate this concept, the expression of CB_1_R and CB_2_R was evaluated on cell surface by means of cytometric analysis and confocal microscopy. As shown in **Fig. 2A**, upon polyclonal stimulation with anti-CD3 and anti-CD28, T-lymphocytes showed a significant increase in CB_1_R and CB_2_R, with the levels of the latter being significantly higher than those of CB_1_R. These effects could be observed in both CD4^+^CD3^+^ and CD8^+^CD3^+^ lymphocytes, and were also confirmed by confocal imaging (**Fig. 2B**), showing that fluorescence intensity of CB_2_R was higher than that of CB_1_R in activated T-cells. In addition, we found that treatment with the CBRs synthetic agonist CP55.940 significantly increased [^35^S]GTPγS binding in activated, but not in resting, T-cells (**Fig. 2C**).

### Effect of AEA on T-cell proliferation and IL-2 production

**Figure 3:**
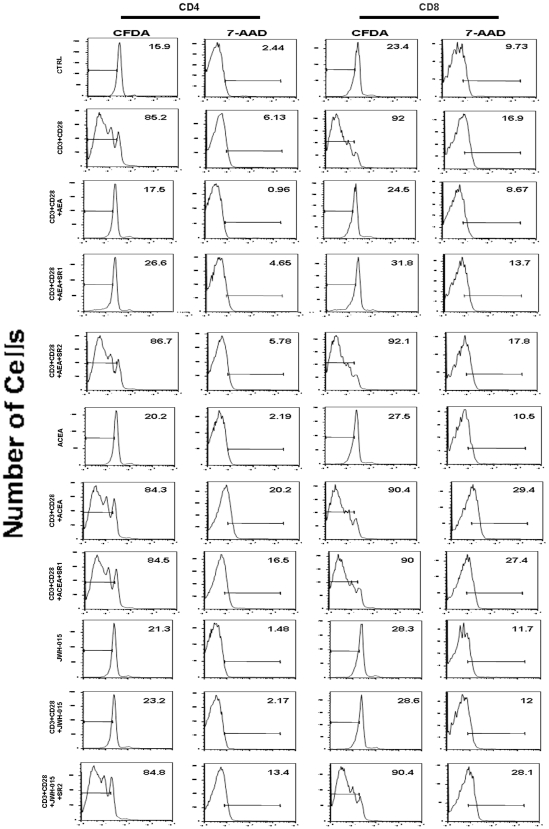
Effect of AEA on cell proliferation *versus* cell viability in anti-CD3/anti-CD28-stimulated T-lymphocytes. Purified resting or CD3/CD28-activated T-lymphocytes (1×10^5^ cells/well) were either left untreated or treated with 2.5 µM AEA and/or cannabinoid receptors agonists or antagonists (each at 1.0 µM). Cells were labelled by means of CFDA-staining as well as by 7-AAD dye, and proliferating cells were analyzed with Flow Cytometry as described in Materials and Methods.

**Figure 4:**
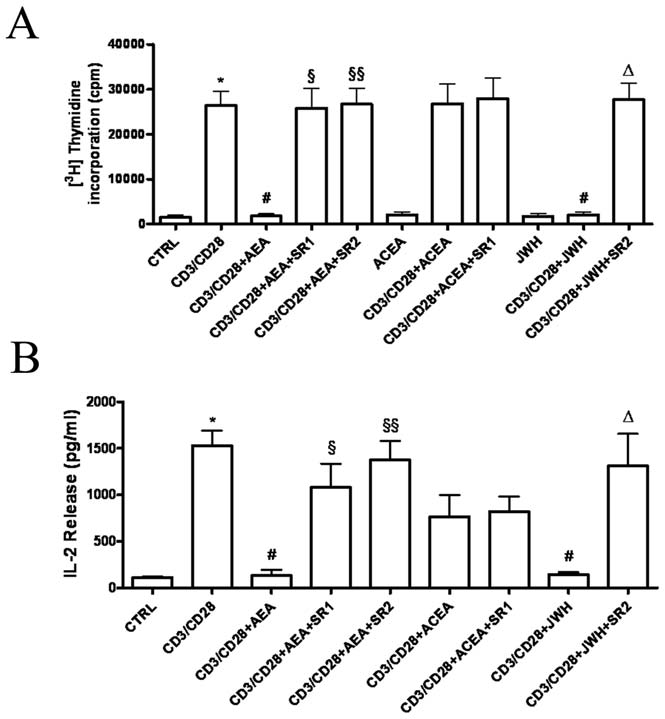
Effect of AEA on IL-2 dependent anti-CD3/anti-CD28-stimulated T-cell proliferation. Purified resting or CD3/CD28-activated T-lymphocytes (1×10^5^ cells/well) were either left untreated or treated with 2.5 µM AEA and/or cannabinoid receptors agonists or antagonists (each at 1.0 µM). **Panel A**: Treated or untreated cells were incubated overnight with [^3^H]-thymidine. Levels of [^3^H]-thymidine incorporation were measured as reported in Materials and Methods. Data were reported as the mean ± SD of 8 independent experiments. * Indicates p<0.001 *versus* CTRL cells, # p<0.01 *versus* CD3/CD28-treated cells, ^§^ p<0.001 *versus* CD3/CD28+AEA-treated cells, and ^Δ^ p<0.01 *versus* CD3/CD28+JWH-015-treated cells. **Panel B**: Supernatants of treated or untreated cells cultured for 24 hours were collected, and IL-2 levels were quantified by means of ELISA. Data are reported as the mean (± SD) of 6 independent experiments. * Indicates p<0.001 *versus* CTRL cells, # p<0.01 *versus* CD3/CD28-treated cells, ^§^ p<0.05 *versus* CD3/CD28+AEA-treated cells, ^§§^ p<0.001 *versus* CD3/CD28+AEA-treated cells, and ^Δ^ p<0.01 *versus* CD3/CD28+JWH-015-treated cells.

Since activated lymphocytes relocalize CB_2_ and (to a lesser extent) CB_1_ receptors, we set out to investigate the effect of CB receptor stimulation on lymphocyte function. In a preliminary set of experiments we tested by cytofluorimetric analysis the effect of different doses of AEA (up to 5.0 µM with 0.5 µM increments) on human T-cell proliferation. We found that AEA dose-dependently reduced cell proliferation, down to a minimum of ∼40% of controls at 2.5 µM, without any further reduction at higher doses (not shown). Therefore, 2.5 µM AEA was chosen to perform all subsequent experiments. First, we measured cell proliferation following polyclonal stimulation, alone or in the presence of the CBR agonist AEA. **Fig. 3** shows that 2.5 µM AEA strongly suppresses anti-CD3/anti-CD28-induced CD4+ and CD8+ T-lymphocyte proliferation. This effect was not due to an induction of necrosis or apoptosis, as assessed by testing cell viability and/or DNA fragmentation through 7-AAD labeling. In addition, the anti-proliferative action of AEA was counteracted by the selective CB_2_R antagonist SR2, used at 1.0 µM, thus suggesting a major involvement of type-2 cannabinoid receptor in this activity of AEA. Such an involvement of CB_2_R was further validated by using the selective CB_2_R agonist JWH-015 (at 1.0 µM), that produced a strong and significant inhibition of T-lymphocytes proliferation. Instead, the CB_1_R selective agonist ACEA was ineffective, when used at 1.0 µM. The anti-proliferative effect of AEA was also confirmed by [^3^H]-thymidine incorporation assays (**Fig. 4A** **)**, in which we demonstrated that AEA significantly inhibited DNA synthesis, and that JWH-015 mimicked this action whereas ACEA did not. Moreover, SR1 or SR2 were able to significantly reduce inhibition of [^3^H]-thymidine incorporation induced by AEA. Incidentally, it should be recalled that AEA is also an endogenous agonist of type-1 vanilloid receptors (TRPV1) [19]. We found that human T lymphocytes express mRNA levels of TRPV1 similar to those of CB_1_R, but they failed to express TRPV1 protein. Consistently, we found that the TRPV1 agonist capsaicin (at doses up to 10 µM) did not affect T-cell proliferation assessed by FACS analysis, and that the TRPV1 antagonist capsazepine (1 µM) did not affect the antiproliferative activity of 2.5 µM AEA (data not shown). Both capsaicin and capsazepine were used at doses previously shown to be effective on the target receptor in immune cells [20]. Furthermore, **Fig. 4B** shows that AEA effectively suppressed the release from T-lymphocytes of the crucial T cell growth factor IL-2 compared to anti-CD3/anti-CD28-treated controls (134±61 pg/ml *versus* 1530±161 pg/ml; n = 9). Again, both SR1 and SR2 minimized this effect of AEA, whereas ACEA was ineffective and JWH-015 strongly reduced IL-2 release (145±24 pg/ml; n = 4). The latter effect was minimized by SR2 (1311±346 pg/ml; n = 4).

### Effect of AEA on cytokine release

**Figure 5:**
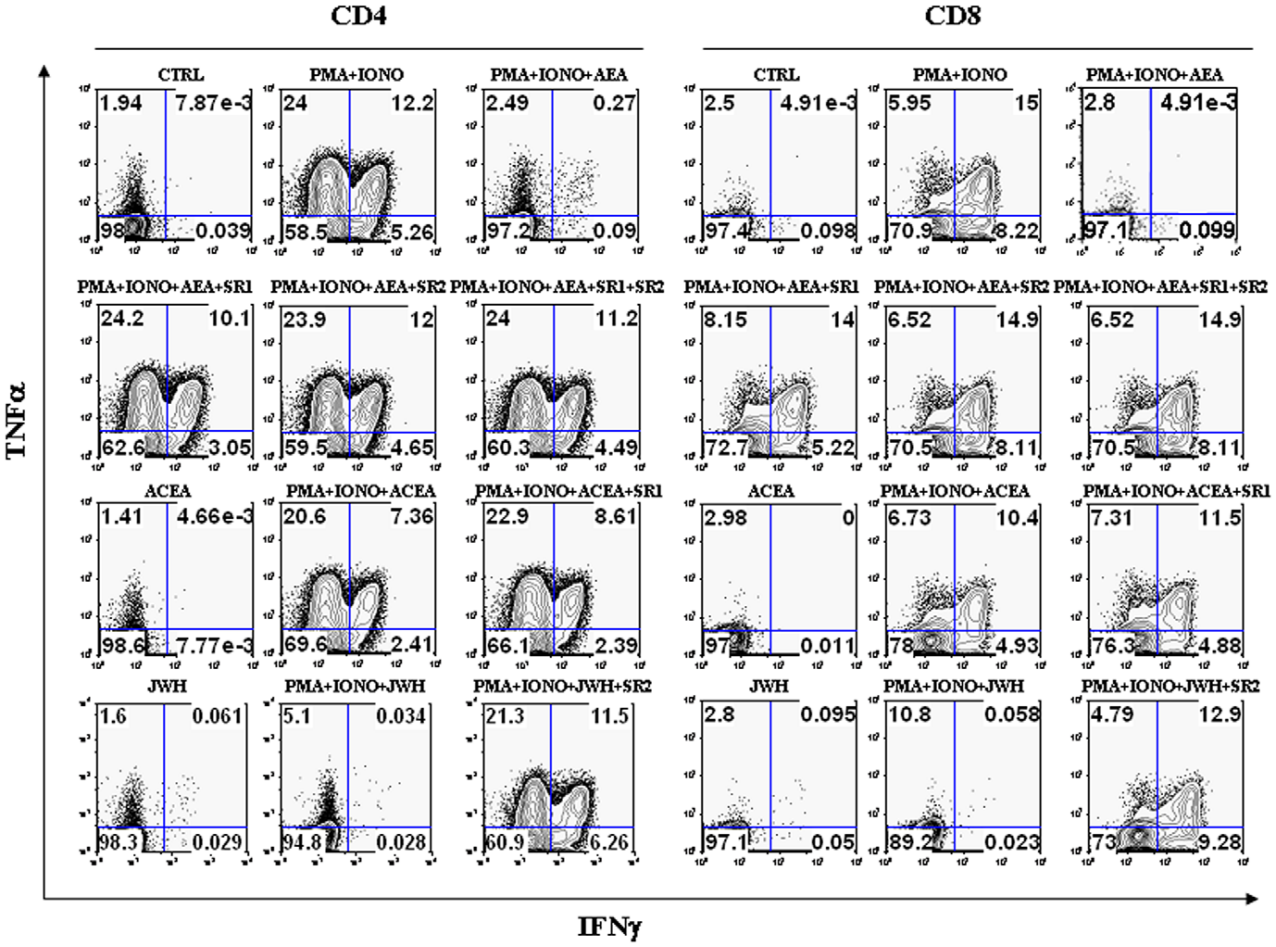
Effect of AEA on TNF-α *versus* IFN-γ production in activated T-lymphocytes. T-lymphocytes (1×10^5^ cells/well) were either left untreated or treated with 2.5 µM AEA and/or cannabinoid receptors agonists or antagonists (each at 1.0 µM). Cells were then stimulated with PMA/ionomycin for 6 hours and stained intracellularly with anti-TNFα-APC and anti-IFNγ-PE. Levels of intracellular cytokine production were analyzed by flow cytometry, as detailed in Materials and Methods, and represent 8 independent experiments.

**Figure 6:**
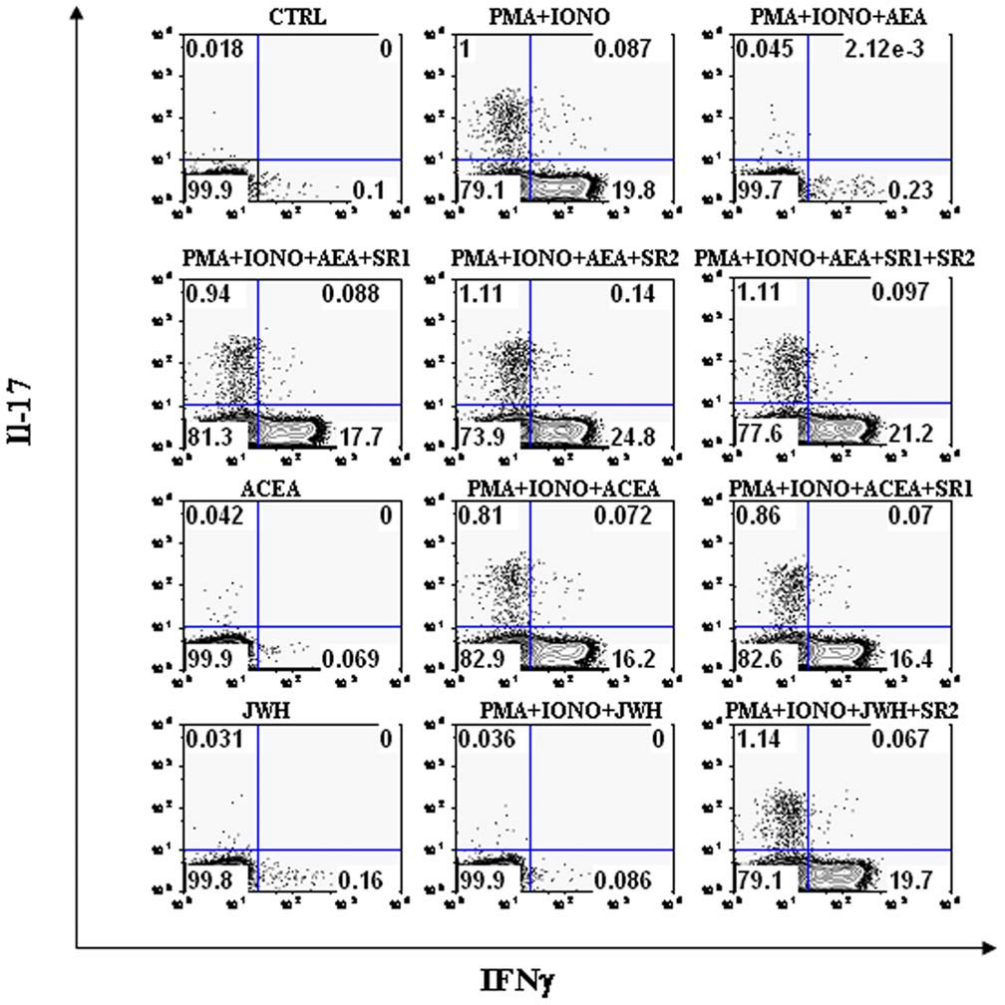
Effect of AEA on IL-17 *versus* IFN-γ production in activated T-lymphocytes. T-lymphocytes (1×10^5^ cells/well) were either left untreated or treated with 2.5 µM AEA and/or cannabinoid receptors agonists or antagonists (each at 1.0 µM). Cells were then stimulated with PMA/ionomycin for 6 hours and stained intracellularly with anti-IL17-PE and anti-IFNγ-APC. Levels of intracellular cytokine production were analyzed by flow cytometry, as detailed in Materials and Methods, and represent 8 independent experiments.

**Figure 7:**
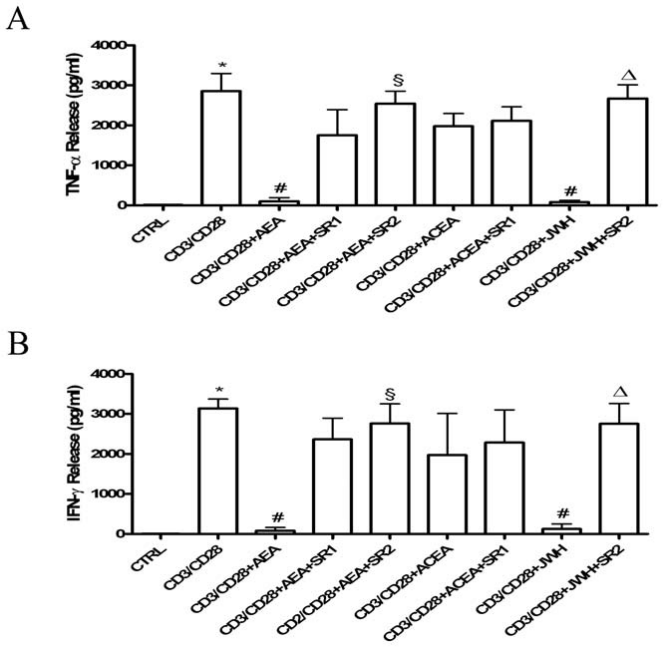
Effect of AEA on cytokine release in anti-CD3/anti-CD28-stimulated T-lymphocytes. Purified resting or CD3/CD28-activated T-lymphocytes (1×10^5^ cells/well) were either left untreated or treated with 2.5 µM AEA and/or cannabinoid receptors agonists or antagonists (each at 1.0 µM). Supernatants of treated or untreated cells cultured for 24 hours were collected, and TNF-α (**Panel A**) and IFN-γ (**Panel B**) levels were quantified by means of ELISA. Data were reported as the mean (± SD) of 6 independent experiments. In panel (**A**), * indicates p<0.01 *versus* CTRL cells, ^#^ p<0.01 *versus* CD3/CD28-treated cells, ^§^ p<0.01 *versus* CD3/CD28+AEA-treated cells, and ^Δ^ p<0.01 *versus* CD3/CD28+JWH-015-treated cells. In panel (**B**), *indicates p<0.01 *versus* CTRL cells, ^#^ p<0.01 *versus* CD3/CD28-treated cells, ^§^ p<0.05 *versus* CD3/CD28+AEA-treated cells, and ^Δ^ p<0.05 *versus* CD3/CD28+JWH-015-treated cells.

Next, we ascertained the possible impact of AEA on the production and release of several cytokines from T-cells. A summary of the amount of cells producing the cytokines analyzed under all experimental conditions tested is reported in **Tables 2** **and** **3**. We found that AEA suppresses TNF-α and IFN-γ production from PMA/ionomycin-activated T-lymphocytes, both in CD4^+^ and CD8^+^ subpopulations (**Fig. 5**). Suppression of TNF-α and IFN-γ production by AEA was significantly counteracted by both CB_1_R and CB_2_R antagonists, but SR2 showed a larger effect than SR1. Moreover, ACEA failed to suppress cytokine production, whereas JWH-015 mimicked the suppressive action of AEA, and SR2 significantly antagonized this effect. Furthermore, the production of IL-17 was also analyzed. In fact, this cytokine is produced only by specialized T-helper cells (Th17), that have been recently recognized as pivotal players in autoimmune diseases and in almost every pro-inflammatory process. As shown in **Fig. 6**, AEA suppressed IL-17 production from PMA/ionomycin-activated CD4+ T-lymphocytes, an effect antagonized by both SR1 and SR2, and was mimicked by JWH-015 but not ACEA. Additionally, **Fig. 6** shows the production of IFN-γ together with that of IL-17, in order to demonstrate that the latter cytokine is produced only by a specific subset of T-cells that, in turn, were unable to produce IFN-γ. Finally, in order to further analyze the effect of AEA on the cytokine profile, a quantitative analysis of TNF-α and IFN-γ content was performed by means of ELISA. **Figs. 7A–B** show that anti-CD3/anti-CD28-activated T-lymphocytes produced significant amounts of TNF-α and IFN-γ (2854±440 pg/ml and 3140±230 pg/ml, respectively; n = 6), whereas in T-cells treated with 2.5 µM AEA the release of cytokines was remarkably inhibited (TNF-α  = 101±91 pg/ml and IFN-γ  =  80±70 pg/ml; n = 6). Interestingly, 1.0 µM SR1 did not significantly antagonize the effect of AEA on cytokine release, whereas 1.0 µM SR2 did (TNF-α  =  2545±311 pg/ml and IFN-γ  =  2761±489 pg/ml; n = 6). In addition, 1.0 µM JWH-015 (TNF-α  =  82.33±41.74 pg/ml and IFN-γ  = 125±115 pg/ml; n = 4), but not 1.0 µM ACEA, mimicked the activity of AEA, and 1.0 µM SR2 counteracted this effect of JWH-015 (TNF-α  =  2673±339 pg/ml and IFN-γ  = 2754±506.5 pg/ml; n = 4).

**Table 2:** Amount of T-lymphocyte subpopulations producing IFN-γ or TNF-α.

	CD4^+^		CD8^+^	
	IFNγ	TNFα	IFNγ	TNFα
**CTRL**	0.03±0.06	0.03±0.04	0.06±0.10	0.02±0.03
**PMA/IONO** a	16.80±4.30*	29.90±7.30*	29.60±8.40*	25.00±9.30*
**PMA/IONO + AEA** b	0.03±0.60^#^	0.49±1.30^#^	1.00±2.70^#^	0.96±1.80^#^
**PMA/IONO + AEA + SR1** c	13.30±3.90^§^	22.80±10.40^§^	21.70±6.90^§^	20.46±9.00^§^
**PMA/IONO + AEA + SR2** d	16.30±5.10^§^	27.90±11.60^§^	26.60±8.60^§^	24.34±9.78^§^
**PMA/IONO + AEA + SR1/SR2**	15.60±3.03^§^	27.36±3.70^§^	26.90±5.90^§^	24.73±9.90^§^
**PMA/IONO + ACEA** e	11.15±0.97	24.60±6.20	21.63±6.16	21.91±11.00
**PMA/IONO + ACEA + SR1**	12.00±1.41	25.92±3.20	21.80±7.30	22.62±9.80
**PMA/IONO + JWH** f	0.02±0.30^#^	2.12±2.10^#^	0.60±0.08^#^	6.40±3.20^#^
**PMA/IONO + JWH + SR2**	16.20±2.80^Δ^	26.68±2.98^Δ^	30.00±7.20^Δ^	23.30±9.20^Δ^

a PMA/IONO  = 1 µM/10 µM.

b AEA  = 2.5 µM.

c SR1  = 1 µM.

d SR2  = 1 µM.

e ACEA  = 1 µM.

f JWH  = 1 µM.

*Denotes p<0.001 *versus* CTRL; # denotes p<0.001 *versus* PMA/IONO; § denotes p<0.001 *versus* PMA/IONO + AEA; ^Δ^ denotes p<0.001 *versus* PMA/IONO + JWH.

**Table 3:** Amount of CD4+ T-lymphocyte subpopulation producing IL-17.

	CD4+IL-17+
**CTRL**	0.06±0.06
**PMA/IONO** a	0.94±0.18*
**PMA/IONO + AEA** b	0.02±0.01^#^
**PMA/IONO + AEA + SR1** c	0.83±0.18^§^
**PMA/IONO + AEA + SR2** d	0.97±0.22^§^
**PMA/IONO+AEA+SR1/SR2**	1.02±0.22^Φ^
**PMA/IONO + ACEA** e	0.76±0.16
**PMA/IONO + ACEA + SR1**	0.77±0.14
**PMA/IONO + JWH** f	0.01±0.01^#^
**PMA/IONO + JWH +SR2**	0.94±0.19^Δ^

a PMA/IONO  = 1 µM/10 µM.

b AEA  = 2.5 µM.

c SR1  = 1 µM.

d SR2  = 1 µM.

e ACEA  = 1 µM.

f JWH  = 1 µM.

*Denotes p<0.01 *versus* CTRL; # denotes p<0.05 *versus* PMA/IONO; § denotes p<0.05 *versus* PMA/IONO + AEA; Φ denotes p<0.001 *versus* PMA/IONO + AEA; ^Δ^ denotes p<0.001 *versus* PMA/IONO + JWH.

## Discussion

Since its identification from porcine brain, AEA has been shown to exert cannabimimetic activities such as the induction of hypothermia, analgesia, and motor effects. Although first described in the nervous system, it is becoming increasingly clear that AEA plays an important role in modulating the immune system. The present study clearly shows that AEA is immunosuppressive when added to activated T-lymphocytes, and that it acts mainly through CB_2_R. In the attempt to shed some light on such immunomodulatory activity of AEA, we first collected evidence for the alteration of the expression of CB_1_R and CB_2_R, as a result of cell activation. In fact, it is well-documented that immune cells, when activated, can modulate specific receptors in order to trigger and sustain an efficient immune response. Our data not only demonstrate by means of several techniques that CB_2_R is by far more abundant than CB_1_R in primary human T-cells, but also that both receptors are recruited on the cell surface upon activation. To date there is evidence showing that classical antigenic stimuli, such as LSP or anti-CD3, can modulate both CB_1_R and CB_2_R expression, however there are discrepancies in the literature especially because of the different experimental models used, spanning from Jurkat cells to mouse splenocytes [8]. Thus, our analysis of CB_1_R and CB_2_R seems to represent the first evidence of a redistribution of CBRs with an increased localization on the cell surface, particularly in the case of CB_2_R. In addition, [^35^S]GTPγS binding data seem to support a receptor “sensitization” in a highly purified population of primary human immune cells, that is CD3^+^-T lymphocytes. This seems of interest, since it allows a better understanding of the role of CB_1_ *versus* CB_2_ receptors in endocannabinoid-mediated immunomodulation of lymphocytes. Increasing evidence supports indeed that both receptors are involved in central nervous system autoimmunity, having particular beneficial effects in the treatment of multiple sclerosis (MS). Using animal models of MS like experimental autoimmune encephalomylelitis (EAE), CB_1_R and CB_2_R have been shown to be specific markers of MS plaque cell subtypes [21]; furthermore, CB_1_R has been proven to mediate suppression of CNS autoimmune inflammation on neurons, whilst CB_2_R had the same anti-inflammatory role on autoreactive T cells [22]. The present results provide evidence that AEA is able to suppress anti-CD3/anti-CD28-induced T-cell proliferation mainly via CB_2_ receptors. In fact, this activity of AEA was minimized by the CB_1_R and CB_2_R antagonists SR1 and SR2, with a major effect exerted by SR2, and was also mirrored by the CB_2_R agonist JWH-015, but not by the CB_1_R agonist ACEA. At any rate, the anti-proliferative action of AEA reported herein is in accordance with a previous report by Schwarz and colleagues [13], where the suppression of proliferation by µM concentrations of AEA was not associated to the induction of apoptosis. Indeed, they demonstrated that AEA is capable of inducing apoptosis only when used at high doses and even that was at least in part responsible for the complete inhibition of cell proliferation observed at high concentrations. The ability of AEA to suppress T-cell proliferation was also substantiated by its ability to markedly inhibit IL-2 release from activated T-lymphocytes, again in a CB_2_R-mediated manner. This result is consistent with a previous report, where inhibition of IL-2 release from phytohemagglutinin-stimulated PBMCs was found to be mediated by CB_2_R [23]. Incidentally, here we could demonstrate that TRPV1 was not engaged in this activity of AEA. In additional experiments, the immunosuppressive effect of AEA was corroborated by a detailed analysis of the production of the major cytokines involved in the regulation of T-lymphocyte responses. Indeed, AEA-induced inhibition of TNF-α and IFN-γ release was predominantly mediated by CB_2_R, since cytokine suppression was not significantly reversed by the CB_1_R antagonist SR1. However, based on the partial effect of the latter compound, and on the fact that specific CB_1_ and CB_2_ ligands like ACEA and JWH-015 can only provide indirect evidence of the involvement of one receptor subtype over the other, a possible contribution of CB_1_R to the activity of AEA cannot be ruled out. In particular, JWH-015 is also a partial agonist of CB_1_R, although with lower affinity than for CB_2_R [24]. In addition, there are reports that even AEA might not act as a physiological agonist of CB_2_R [25], although this endocannabinoid has been shown to exert manifold CB_2_R-dependent activities in experimental paradigms, and more recently its localization within biological membranes has led to a reconsideration of its role as a true CB_2_R agonist *in vivo* [26], [27]. At any rate, it should be stressed that there are several studies documenting alterations in cytokines release induced by endocannabinoids on immune cells [28], [29], but none of them has ever been performed on primary human T-lymphocytes. The capacity of anandamide to suppress the proinflammatory response of T-cells is of pivotal importance, because not only it implies a role in inhibiting IFN-γ-mediated T-helper 1 (Th-1) responses, but it could also suggest a potential down-stream effect of this endocannabinoid also in modulating the cross-talk between T-lymphocytes and several other immune cells, including B-cells, macrophages and neutrophils. Activation of these cells is crucial in several immune-mediated diseases, and in most recent years a minor subset of T cells has gained centre stage in the study of the pathogenesis of immune disorders. These cells produce IL-17, a potent cytokine which contributes to host defense against extracellular pathogens and which has been clearly shown to be involved in the development of autoimmune diseases [30]. Current research is investigating the possibility to interfere with the function of these cells, and the finding that a natural endogenous compound such as anandamide exerts a suppressive – but not cytotoxic – effect also on cells with a central role in the induction of autoimmunity, represents a promising beginning for a new avenue of research. It should be underlined that most immunosuppressive therapies involve the use of compounds which are cytotoxic for T-cells, thus exposing the patients to increased risk of infections. The finding that AEA preserves cell viability whilst containing the proinflammatory response represents an innovative approach in the effort to avoid autoimmune reactivity without affecting protective immune responses. On this basis, the present evidence for an immunosuppressive effect of AEA also on IL-17 production seems very timely, and is suggestive of new therapeutic approaches that could potentially target autoimmune diseases.
